# Differentiating Kawasaki Disease and Multisystem Inflammatory Syndrome in Children Using Blood Composite Scores: Insights into Clinical Outcomes and Predictive Indices

**DOI:** 10.1007/s00246-025-03785-w

**Published:** 2025-01-28

**Authors:** Kadir Ulu, Şengül Çağlayan, Taner Coşkuner, Ebru Oğultekin Vazgeçer, Taliha Öner, Betül Sözeri

**Affiliations:** 1 Department of Pediatric Rheumatology, Şehit Prof. Dr. İlhan Varank Sancaktepe Training and Research Hospital, University of Health Sciences, Emek-Namık Kemal Avenue No. 54, 34785 Sancaktepe, Istanbul, Turkey; 2 Department of Pediatric Rheumatology, Ümraniye Training and Research Hospital, University of Health Sciences, Istanbul, Turkey; 3 Department of Social Pediatrics, Ümraniye Training and Research Hospital, University of Health Sciences, Istanbul, Turkey; 4 Department of Pediatric Cardiology, Ümraniye Training and Research Hospital, University of Health Sciences, Istanbul, Turkey

**Keywords:** Kawasaki disease, MIS-C, Composite score, Inflammatory indices

## Abstract

The study sought to assess the clinical utility of complete blood count-derived composite scores, suggesting their potential as markers of inflammation and disease severity in Kawasaki disease (KD) and multisystem inflammatory syndrome in children (MIS-C) with Kawasaki-like features. This retrospective study analyzed data from 71 KD and 73 MIS-C patients and 70 healthy controls. The KD group showed a higher rate of coronary involvement (26.7% vs. 10.9%), while the MIS-C group had a higher intensive care unit (ICU) admission rate (34.2% vs. 2.8%). Platelet counts, lymphocyte counts, mean platelet volume (MPV), MPV/Lymphocyte (MPVLR), and MPV/Platelet (MPVPR) ratios demonstrated the highest specificities in distinguishing MIS-C than KD (84.5%, 83.1%, 91.1%, 88.7%, and 88.7%, respectively). Monocyte counts, MPV, and MPVPR demonstrated the highest specificities to predictive ICU admission in the MIS-C group (83.3%, 89.6%, and 89.6%, respectively). Lymphocyte counts, platelet/lymphocyte ratio (PLR), neutrophil/lymphocyte ratio (NLR), MPVLR, and Systemic Immune-Inflammation Index (SII) parameters were found to have high negative predictive values for predicting KD patients without coronary artery lesions (CALs) (85.7%, 86.1%, 87.1%, 87.1%, and 85.7%, respectively)., Systemic Inflammation Response Index (SIRI), MPVPR, and CRP were independently predictive of ICU admission in the MIS-C group, and lymphocyte count and IVIG resistance were also identified as significant predictors of CALs in the KD group. NLR, MPVLR, MPVPR, and NPR indices effectively differentiate MIS-C from KD and predict ICU admission in MIS-C. NLR, PLR, MPVLR, and SII are valuable in excluding CALs in KD with high negative predictive values. In addition, SIRI and MPVLR were independent predictors of ICU admission in MIS-C, and lymphocyte count was identified as an independent predictor of CALs in KD.

## Introduction

In April 2020, a novel syndrome characterized by severe multi-system inflammation in children was described several weeks after exposure to SARS-CoV-2 [1, 2]. Multisystem inflammatory syndrome in children (MIS-C) shares clinical features with infectious and inflammatory diseases, making differentiation challenging. MIS-C is a complex disease that can cause macrophage activation syndrome and shock. A cluster study showed that one-third of MIS-C patients exhibit Kawasaki-like features [3–5]. Kawasaki disease (KD) is a leading cause of acquired heart disease in children, characterized by systemic vasculitis. Although the relationship between common respiratory viruses, including coronaviruses, and the development of KD has been previously reported, the etiological agents remain to be elucidated [6–8]. It is hypothesized that an infectious agent triggers innate and adaptive immune responses in genetically susceptible children [2]. Both conditions appear to share inflammatory features associated with post-infectious hyperinflammatory processes.

Multisystem inflammatory syndrome in children is characterized by dysregulated cellular and humoral immune responses, including reductions in plasmacytoid dendritic cells, monocytes, and natural killer cells [9, 10]. The role of platelet increase in the pathogenesis and severity of KD was described [11]. In both entities, spontaneous release of neutrophil extracellular traps by neutrophils may be associated with vascular damage [12, 13]. Compared to KD, cases with MIS-C exhibit lower lymphocyte and platelet counts. The elevation of various autoantibodies and cytokines, including IL-10, TNF-α, IFN-γ, and IL-1β, has been documented in both conditions. Additionally, some, such as IL-17A, have been reported to be elevated to varying degrees [2, 10].

Simple and accessible tests could help clinicians diagnose accurately and tailor treatments. There has been significant interest in peripheral blood-cell-derived inflammation indices in recent years. The indices offer insights into the balance between pro-inflammatory and anti-inflammatory responses within the body. A considerable body of research has been conducted to examine the impact of these markers on disease activity, mortality, and prognosis [14–23]. The predictive value of these markers, including the Systemic Immune-Inflammation Index (SII), Systemic Inflammation Response Index (SIRI), Pan-Immune-Inflammation Value (PIV), platelet/lymphocyte ratio (PLR), and neutrophil/lymphocyte ratio (NLR), is increasingly recognized in medical research. These markers are valuable in clinical settings as they are derived from routine blood tests, making them accessible and cost-effective tools for monitoring inflammation and guiding treatment strategies. At present, there is a lack of studies that provide a detailed comparison of inflammatory markers in KD and MIS-C with Kawasaki-like features. The aim of this study is to distinguish between two phenotypically similar diseases by analyzing immunopathogenic differences reflected in complete blood counts. Furthermore, we sought to demonstrate the relationship between the clinical outcomes and the blood composite scores.

## Method

### Study Population

This retrospective study included patients with KD who were followed up between June 2016 and January 2023 and patients with MIS-C who were followed up between March 2020 and January 2023 at Ümraniye Training and Research Hospital. Patients with MIS-C were diagnosed based on criteria recommended by the Centers for Disease Control and Prevention and the World Health Organization [24, 25]. The diagnosis of KD was made according to the definition provided by the American Heart Association [26]. The data of 75 patients with KD and 195 patients with MIS-C were extracted from medical records or electronic data systems for analysis. Patients diagnosed with KD and MIS-C were evaluated by an expert pediatric rheumatologist (BS) using established definitions and criteria. MIS-C patients who exhibited at least two major clinical features of KD (such as rash, changes in oral mucosa, bilateral conjunctival injection, cervical lymphadenopathy, and erythema or edema of the extremities) were classified as having "MIS-C with KD-like features". Patients diagnosed with MIS-C who did not have these features were excluded. We conducted a thorough review of medical records, encompassing clinical assessments, laboratory results, and echocardiographic data. To ensure accurate diagnoses during the pandemic, we confirmed negative PCR and serology results for all KD patients, minimizing the possibility of misdiagnosis. This rigorous and methodical approach allowed for a clear separation of MIS-C and KD, enabling precise comparative analysis. Of the patients diagnosed with MIS-C, 78 exhibited Kawasaki-like features. Nine patients (4 KD and 5 MIS-C) were excluded due to referrals from other hospitals, incomplete data, or prior medication use before admission. Healthy controls were randomly selected from children who attended routine health check-ups in general pediatrics and social pediatrics outpatient clinics and had a complete blood count in their medical records. Those with any history of chronic illness, infection, or inflammation were excluded.

### Study Design and Data Collection

A standardized form was employed to record patients' demographic data, clinical characteristics, and laboratory findings upon admission. SII was calculated as platelet × neutrophil/lymphocyte, SIRI as neutrophil × monocyte/lymphocyte, and PIV as platelet × neutrophil × monocyte/lymphocyte as reported in previous studies. In addition, as in previously reported studies, NLR, PLR, monocyte/lymphocyte Mean platelet volume/platelet (MPVPR), MPV/lymphocyte (MPVLR), neutrophil/platelet × 100 counts (NPR) ratios were calculated [14–23]. The demographic, clinical, and laboratory characteristics, along with inflammatory indices, were compared among the KD, MIS-C, and healthy control groups. The predictive value of composite scores for coronary artery lesions (CALs) in KD and intensive care unit (ICU) admission in MIS-C was assessed, alongside their correlation with clinical outcomes.

### Ethics

Ethical approval was obtained from the local ethics committee (Date: July 11, 2024, No: B.10.1.TKH.4.34.H.GP.0.01/220). The study was performed according to the tenets of the Declaration of Helsinki. Due to its retrospective nature, the requirement for informed consent from participants was waived.

### Statistical Analysis

The analysis was conducted using IBM SPSS version 30.0.0.0. The graphs were created using GraphPad Prism, version 10.3.1. The Skewness-Kurtosis tests were used to check the normality of numerical variables. Descriptive statistics were presented as median [interquartile range (IQR)] and percentages. Fisher’s exact or chi-square tests were used for categorical variables, as appropriate. Pairwise comparisons for scale data were performed using the Mann–Whitney U test and independent sample t-test, as applicable. Pearson and Spearman correlation tests were used based on suitability. Receiver operating characteristic (ROC) curves were used to evaluate the overall discriminatory ability of inflammatory indices. For ROC analysis, we focused on area under the curve (AUC) values greater than 0.70 or p values less than 0.01. Youden Index was calculated as sensitivity + 1-specificity. Binary logistic regression analysis using the forward likelihood ratio method identified the independent predictors of the outcome variable. We assessed potential multicollinearity using the Variance Inflation Factor, retaining only those with a VIF below 10 for the final model. Significant predictors were reported with their Exp(B) values and 95% confidence intervals.

## Results

### General Characteristics of the Study Population

A total of 144 (F/M: 60/84) patients, including 71 KD and 73 MISC with Kawasaki-like findings, and 70 (F/M: 34/36) healthy controls, were included in the study. Patients with KD had a younger median age (6.5 vs. 7.4 years) and age at diagnosis (2.5 vs. 5.7 years) than MIS-C subjects (*p* = 0.008, *p* < 0.001). There was no significant difference between the control and patient groups regarding gender (*p* = 0.460). Regarding age at the time of obtaining complete blood count data, the healthy group and the KD group had similar ages (2.5 and 2.3 years) (*p* = 0.529), while the MIS-C group was older (5.7 years) (*p* < 0.001). The KD group showed a higher rate of coronary involvement (26.7% vs. 10.9%) (*p* = 0.003), while the MIS-C group had a higher ICU admission rate (34.2% vs. 2.8%) (*p* < 0.001). The median length of stay in the ICU for these patients was 4 (Q1, Q3: 2–7) days. Conjunctivitis (77.5 vs. 68.5%), lymphadenopathy (73.2 vs. 41.1%), mucosal changes (83.1 vs. 57.5%), edema of the extremities (71.8 vs. 20.5%) were more common in the KD group than in the MIS-C group. In contrast, macular rash (57.7 vs. 63%) was relatively less common. The clinical and demographic characteristics of the groups are presented in Table 1.

**Table 1:** Clinical and demographic characteristics, laboratory findings, and inflammatory indices in Kawasaki disease, multisystem inflammatory syndrome in children, and healthy controls

	KD	MIS-C	Healthy controls	p^1^	p^2^	p^3^
Female/Male	31/40	29/44	34/36	0.566^†^		
Age, years ^#^	2.5(1.5–4.2)	5.67(3.75–11)	2.25(1.52–4.02)	** < 0.001**	0.529	** < 0.001**
Length of stay, days	8(6–11)	8(6–11)		0.885		
Fever duration, days	7(5–8)	5(4–7)		** < 0.001**		
Diagnosis delay, days	6(5–7)	5(4–7)		0.070		
Conjunctivitis	55(77.5)	50(68.5)		0.306		
Lymphadenopathy	52(73.2)	30(41.1)		** < 0.001**		
Macular rash	41(57.7)	46(63)		0.518		
Mucosal change	59(83.1)	42(57.5)		** < 0.001**		
Edema at the extremities	51(71.8)	15(20.5)		** < 0.001**		
CALs	19(26.7)	8(10.9)		**0.003**		
ICU admission	2(2.8)	25(34.2)		** < 0.001**		
CRP, mg/dl	8.5(4.9–12.4)	9.36(5.1–14.7)		0.217		
Albumin, g/dl	3.7(3.4–4)	3.27(2.91–3.72)		** < 0.001**		
WBC, 10^3^/µL	14.4(11.2–17.47)	11.24(7.68–15.66)	8.9(7.37–10.93)	**0.030**	** < 0.001**	** < 0.001**
Neutrophil, 10^3^/µL	9(6.97–11.13)	9.06(6.17–12.22)	2.71(2.3–3.84)	0.708	** < 0.001**	** < 0.001**
Lymphocyte, 10^3^/µL	3.82(2.6–5.32)	1.41(0.71–2.41)	5.28(3.98–6.68)	** < 0.001**	** < 0.001**	** < 0.001**
Monocyte, 10^3^/µL	0.93(0.67–1.14)	0.45(0.23–0.75)	0.57(0.46–0.71)	** < 0.001**	** < 0.001**	**0.028**
Platelet, 10^3^/µL	408(330–494)	214(163–283)	301(280–348)	** < 0.001**	** < 0.001**	** < 0.001**
MPV, fl	8(7.34–8.4)	9.1(8.3–10.3)	8.4(7.8–9)	** < 0.001**	** < 0.001**	** < 0.001**
Hemoglobin, g/dl	10.8(9.8–11.6)	11.4(10.3–12.4)	11.3(10.1–12.2)	**0.006**	0.251	0.295
NLR	2.51(1.65–3.69)	7.02(2.85–13.76)	0.56(0.4–0.83)	** < 0.001**	** < 0.001**	** < 0.001**
PLR	100.6(84.3–147.8)	158.7(96.8–268.6)	59.3(46.8–82.5)	** < 0.001**	** < 0.001**	** < 0.001**
MLR	0.23(0.18–0.35)	0.33(0.21–0.49)	0.12(0.09–0.13)	**0.001**	** < 0.001**	** < 0.001**
MPVLR	1.99(1.54–2.77)	6.4(3.4–13.77)	1.61(1.23–2.13)	** < 0.001**	**0.005**	** < 0.001**
MPVPR	0.019(0.016–0.024)	0.043(0.029–0.061)	0.028(0.024–0.032)	** < 0.001**	** < 0.001**	** < 0.001**
NPR	2.15(1.62–3.04)	4.39(2.86–5.94)	0.92(0.69–1.31)	** < 0.001**	** < 0.001**	** < 0.001**
SII	1016.8(728.9–1371.2)	1377.9(688.4–2660.6)	165.9(124.2–253.1)	**0.014**	** < 0.001**	** < 0.001**
SIRI	2.26(1.38–3.57)	3.1(1.66–5.17)	0.3(0.22–0.55)	0.054	** < 0.001**	** < 0.001**
PIV	960.7(550.9–1504)	711.7(241.5–1246.9)	92.2(63.9–171.2)	0.092	** < 0.001**	** < 0.001**

The data are expressed as n (%), median (Q1-Q3) values

Bold values indicate statistical significance

p^1^: KD vs. MIS-C, p^2^: KD vs. Healthy controls p^3^: MIS-C vs. Healthy controls

^#^ The median age at the time of complete blood count was provided

^†^ The value indicates the chi-square analysis between three groups

*CRP* C-reactive protein, *ICU* Intensive care unit, *KD* Kawasaki Disease, *MIS-C* Multisystem inflammatory syndrome in children, *MLR* Monocyte/lymphocyte ratio, *MPVLR* Mean platelet volume/ lymphocyte ratio, *MPVPR* Mean platelet volume/ Platelet ratio, *MPV* Mean platelet volume, *NLR* Neutrophil/lymphocyte ratio, *NPR* Neutrophil/ platelet ratio, *PIV* Pan-immune-inflammation value, *PLR* Platelet/lymphocyte ratio, *SII* Systemic immune-inflammation Index, *SIRI* Systemic inflammatory response index, *WBC* White blood cell

### Comparison of Hematological Parameters and Composite Scores Between Kawasaki Disease, Multisystem Inflammatory Syndrome in Children, and Healthy Controls

A comparison of the healthy group with the MIS-C and KD groups revealed that all inflammatory indices were significantly lower in the former, except MPVPR (*p* values < 0.001). MPVPR was lower in the healthy group than in the MIS-C group (0.028 vs. 0.043) and higher in the KD group (0.028 vs. 0.019), with *p* values < 0.001. In the MIS-C group compared to the KD group, the median values for platelet (214 vs. 408 × 10^3^/µL), lymphocyte (1.41 vs. 3.82 × 10^3^/µL), monocyte (0.45 vs. 0.93 × 10^3^/µL), and albumin (3.27 vs. 3.70 g/dL) were significantly lower, with *p* values < 0.001. Conversely, the median MPV was higher in the MIS-C group (9.1 vs. 8.0 fl) (*p* < 0.001). Compared to the KD group, the MIS-C group exhibited elevated levels of inflammatory indices, apart from PIV. A notable discrepancy was observed between the two groups regarding NLR, PLR, MLR, NPR, SII, MPVLR, and MPVPR median levels with *p* values < 0.05. Laboratory findings and inflammatory indices of the groups are presented in Table 1.

**Fig. 1:**
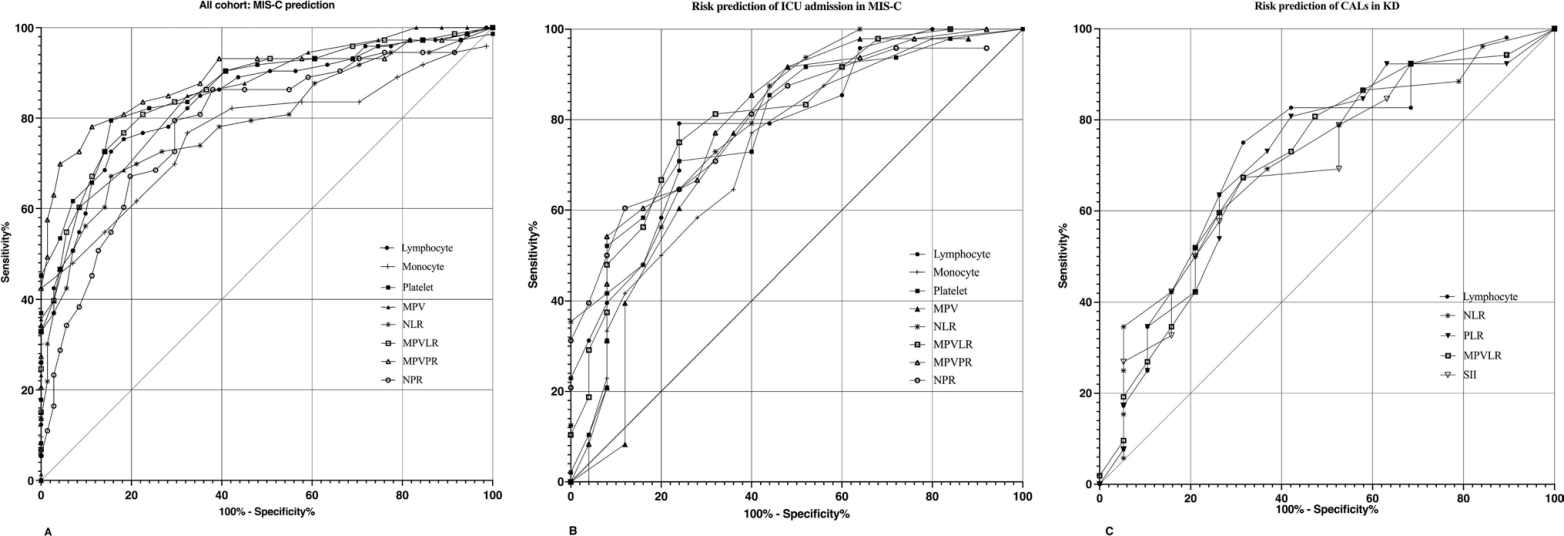
Receiver operating characteristic curves for the prediction of multisystem inflammatory syndrome in children and Kawasaki disease **A** the prediction of admission to the intensive care unit in patients with multisystem inflammatory syndrome in children **B** and the prediction of coronary artery lesions in patients with Kawasaki disease **C**

No standardized laboratory reference values were identified; therefore, we conducted ROC curve analyses to determine the optimal threshold values. Platelet levels (AUC: 0.866) with a cut-off of 294.5 × 10^3^/µL or lower, lymphocyte levels (AUC: 0.841) with a cut-off of 2.31 × 10^3^/µL or lower, MPV (AUC: 0.845) with a cut-off of 8.85 or higher, MPVLR (AUC: 0.862) with a cut-off of 3.88 or higher, and MPVPR (AUC: 0.885) with a cut-off of 0.029 or higher demonstrated the highest specificities (84.5%, 83.1%, 91.1%, 88.7%, and 88.7%, respectively) in distinguishing MIS-C than KD. The ROC curves and analysis results for the most critical parameters used to differentiate between the two diseases are presented in Fig. 1 and Table 2.

**Table 2:** Receiver operating characteristic analysis of diagnostic and prognostic parameters in MIS-C and KD

All cohort: MIS-C prediction								
Parameters	Cut-off	AUC	95% CI	Sensitivity, %	Specificity, %	PPV, %	NPV, %	p
Lymphocyte	< 2.31	841	776–906	74	83.1	81.8	75.6	< 0.001
Monocyte	< 0.75	767	688–847	75.3	70.4	72.4	73.5	< 0.001
Platelet	< 294.5	866	805–926	79.5	84.5	84.1	80	< 0.001
MPV	> 8.85	845	783–907	64.4	91.1	87	71.1	< 0.001
NLR	> 3.86	785	710–861	69.9	80.3	77.3	71.8	< 0.001
MPVLR	> 3.88	862	802–922	72.6	88.7	86.9	75.9	< 0.001
MPVPR	> 0.029	885	829–942	78.1	88.7	87.7	79.7	< 0.001
NPR	> 2.68	785	710–862	80.8	70.4	73.8	78.1	< 0.001
MIS-C Group: ICU admission risk prediction								
Lymphocyte	< 1.09	788	679–896	76	79.2	65.5	86.4	< 0.001
Monocyte	< 0.25	735	610–860	60	83.3	65.2	80	0.001
Platelet	< 208	782	667–897	80	70.8	58.8	87.2	< 0.001
MPV	> 10.1	765	634–895	64	89.6	76.2	82.7	< 0.001
NLR	> 7.28	810	707–914	76	68.7	55.9	85.6	< 0.001
MPVLR	> 7.09	812	707–915	80	75	62.5	87.8	< 0.001
MPVPR	> 0.058	791	674–907	60	89.6	75	81.1	< 0.001
NPR	> 3.94	802	702–901	88	60.4	53.7	90.6	< 0.001
KD Group: CALs risk prediction								
Lymphocyte	> 4.33	728	592–865	63.2	80.8	54.5	85.7	0.003
NLR	< 1.95	707	574–841	73.7	59.6	40	86.1	0.007
PLR	< 93.5	694	568–847	73.7	65.4	43.7	87.1	0.007
MPVLR	< 1.88	709	555–833	73.7	67.3	45.1	87.5	0.012
SII	< 858	709	568–849	68.4	69.2	44.8	85.7	0.007

*CALs* Coronary artery lesions, *KD* Kawasaki Disease, *ICU* Intensive CARE Unit, *IVIG* Intravenous immunoglobulin, *MIS-C* Multisystem inflammatory syndrome in children, *MPVLR* Mean platelet volume/lymphocyte ratio, *MPVPR* Mean platelet volume/platelet ratio, *NLR* neutrophil/lymphocyte ratio, *NPR* Neutrophil/platelet ratio, *NPV* Negative predictive value, *PPV* Positive predictive value, *SII* Systemic Immune-inflammation index

### Association of Hematological Parameters and Composite Scores with Clinical Outcomes in Subgroups

In patients with MIS-C, the median values of NLR, PLR, MPVLR, MPVPR, NPR, and SII were higher in patients requiring ICU than those not (*p* values < 0.05). Comparative data of patients requiring intensive care compared to those without are presented in Table 3. Monocyte counts (AUC: 0.735) with a cut-off of 0.25 × 10^3^/µL or lower, MPV (AUC: 0.765) with a cut-off of 10.1 or higher, and MPVPR (AUC: 0.791) with a cut-off of 0.058 or higher demonstrated the highest specificities (83.3%, 89.6%, and 89.6%, respectively) to predictive ICU admission (Fig. 1, Table 2). Moreover, the multivariate regression analysis demonstrated that SIRI (OR: 1.24, 95% CI: 1.07–1.44), MPVPR (OR: 1.69, 95% CI: 1.30–2.19), and CRP (OR: 1.06, 95% CI: 1.01–1.17) were independently predictive factors for ICU admission (Table 4). A moderate positive correlation was observed between length of hospital stay and NLR, MLR, MPVLR, and NPR, with p values of 0.01. Additionally, a moderate negative correlation was evident between fever duration and MPVLR and MPVPR, with p values of 0.01 (Table 5).

**Table 3:** Laboratory findings and inflammatory indices in Kawasaki Disease patients (With CALs vs. without CALs) and in MIS-C patients (ICU vs. Non-ICU)

	KD			MIS-C		
	CALs, n:19	No CALs, n:52	p	ICU admission, n:25	Non-ICU, n:48	p
Age, years ^#^	1.9(0.6–3.1)	2.58(1.59–4.6)	0.073	9.67(5.5–11.75)	4.5(2.25–9.21)	< 0.001
Diagnosis delay, days	7(4–10)	5(5–7)	0.542	4(3–6)	5(4–7)	0.142
WBC, 10^3^/µL	14.6(12.36–18.73)	14.25(10.62–17.47)	0.863	10.02(7.46–14.3)	11.39(8.02–15.69)	0.795
Neutrophil, 10^3^/µL	8.97(8–9.8)	9.13(6.85–11.97)	0.151	9.06(6.5–12.53)	8.97(6.09–11.22)	0.295
Lymphocyte, 10^3^/µL	5.2(3.72–6.6)	3.67(2.51–4.43)	**0.007**	0.76(0.37–1.08)	1.83(1.15–3.18)	** < 0.001**
Monocyte, 10^3^/µL	0.98(0.8–1.18)	0.89(0.62–1.07)	0.364	0.23(0.19–0.5)	0.6(0.28–0.86)	** < 0.001**
Platelet, 10^3^/µL	443(362–489)	376.5(323–494)	0.471	169(107–206)	246(192.5–319)	** < 0.001**
MPV, fl	8.05(7.6–8.5)	8(7.2–8.3)	0.425	10.4(9.1–11.4)	8.9(8.2–9.65)	** < 0.001**
Hemoglobin, g/dl	10.8(9.8–11.9)	10.8(9.9–11.55)	0.948	12(10.3–12.7)	11.3(10.45–12.2)	0.266
NLR	1.88(1.27–2.62)	2.63(2.02–4.07)	**0.008**	14.36(7.4–23.99)	4.82(2.18–8.36)	** < 0.001**
PLR	89.8(59.3–106.9)	111.7(86.7–161)	**0.013**	249.9(122.1–320)	134.4(91.2–217.7)	**0.008**
MLR	0.25(0.19–0.35)	0.21(0.15–0.26)	0.155	0.33 (0.19–0.45)	0.43(0.24–0.85)	**0.014**
MPVLR	1.56(1.21–2.28)	2.05(1.77–3.04)	**0.007**	12.99(7.49–24.87)	4.78(2.61–7.13)	** < 0.001**
MPVPR	0.019(0.017–0.024)	0.019(0.016–0.026)	0.502	0.066(0.044–0.101)	0.035(0.027–0.049)	** < 0.001**
NPR	2.15(1.34–2.62)	2.19(1.64–3.1)	0.154	5.94(4.4–8.17)	3.47(2.41–4.77)	** < 0.001**
SII	718.5(540.9–1034.9)	1048.8(806.6–1452.8)	**0.007**	2470.1(1097.9–3885.5)	1114.1(608.3–2187.6)	**0.007**
SIRI	1.9(1.38–3.41)	2.49(1.36–3.9)	0.104	3.75(2.24–7.49)	2.63(1.5–4.86)	0.066
PIV	779.7(421.7–962)	992.4(661.3–1509.4)	0.102	711.9(241.5–1402.3)	676.6(242.6–1200.2)	0.852
CRP, mg/dl	7.9(2.6–12.4)	9.2(5.15–13.1)	0.140	12.96(2.94–20.52)	8.02(5.15–13.86)	0.085
Albumin, g/dl	3.7(3.5–4.1)	3.6(3.4–4)	0.447	3.09(2.73–3.36)	3.53(3.07–3.81)	**0.003**

The data are expressed as median (Q1-Q3) values. Bold values indicate statistical significance

*CALs* Coronary artery lesions, *CRP* C-reactive protein, *ESR* Erythrocyte sedimentation rate, *ICU* Intensive care unit, *KD* Kawasaki Disease, *MIS-C* Multisystem inflammatory syndrome in children, *MLR* Monocyte/lymphocyte ratio, *MPVLR* Mean Platelet volume/ lymphocyte ratio, *MPVPR* Mean Platelet volume/ Platelet ratio, *MPV* Mean platelet volume, *NLR* Neutrophil/lymphocyte ratio, *NPR* Neutrophil/ platelet ratio, *PIV* Pan-Immune-inflammation value, *PLR* Platelet/lymphocyte ratio, *SII* Systemic IMMUNE-inflammation index, *SIRI* Systemic inflammatory response index, *WBC* White blood cell

**Table 4:** Results from binary logistic regression analysis

Independent factors: For ICU admission in MIS-C patients						
	Univariate			Multivariate		
	Exp(B)	95% CI	p	Exp(B)	95% CI	p
SIRI	1.14	1.03–1.30	0.028	1.24	1.07–1.44	0.004
MPVPR	1.47	1.48–1.83	< 0.001	1.69	1.30–2.19	< 0.001
CRP	1.06	1.01–1.12	0.028	1.06	1.01–1.17	0.044
NPR	1.62	1.25–2.09	< 0.001			
Lymphocyte	0.31	0.14–0.63	0.001			
Albumin	0.22	0.08–0.64	0.005			
Age at Diagnosis	1.19	1.06–1.34	0.003			
Independent factors: For CALs in KD patients						
	Univariate			Multivariate		
Lymphocyte	1.58	1.14–2.19	0.006	1.56	1.11–2.21	0.010
IVIG-resistance	6.41	1.35–30.38	0.019	6.26	1.07–36.65	0.042

CALs: Coronary artery lesions, KD: Kawasaki Disease, ICU: Intensive Care Unit, CRP: C-reactive protein, MIS-C: Multisystem Inflammatory Syndrome in Children, MPVPR: Mean Platelet Volume/Platelet Ratio, NLR: Neutrophil/Lymphocyte Ratio, NPR: Neutrophil/Platelet Ratio, SIRI: Systemic Inflammatory Response Index

**Table 5:** Coefficients for correlations between laboratory findings, inflammatory indices and fever duration, length of stay in KD and MIS-C patients

	KD		MIS-C	
	Length of Stay	Duration of fever	Length of Stay	Duration of fever
WBC	0.021	0.068	0.054	0.386**
Neutrophil	0.055	− 0.038	0.166	0.330**
Lymphocyte	0.028	0.310**	− 0.384**	0.389**
Monocyte	− 0.087	− 0.104	− 0.301**	0.259*
Platelet	0.180	0.270*	− 0.368**	0.327**
MPV	− 0.122	− 0.280*	0.290*	− 0.237
NLR	0.083	− 0.195	0.441**	− 0.131
PLR	0.108	− 0.099	0.273*	− 0.208
MLR	− 0.005	− 0.278*	0.345**	0.042
MPVLR	0.043	− 0.247*	0.393**	−0.430**
MPVPR	− 0.134	− 0.266	0.272*	−0.310**
NPR	− 0.016	− 0.168	0.380**	−0.034
SII	0.171	− 0.076	0.280*	0.080
SIRI	− 0.011	− 0.237*	0.291*	0.142
PIV	0.105	− 0.097	0.054	0.265*
CRP	0.013	0.052	0.171	0.018
Albumin	− 0.312**	− 0.132	− 0.235*	− 0.171

^**^. Correlation is significant at the 0.01 level. *. Correlation is significant at the 0.05 level

Pearson (R) is used for normally distributed variables, and Spearman (Rho) for non-normally distributed variables

*CRP* C-reactive protein, *KD* Kawasaki Disease, *MIS-C* Multisystem Inflammatory syndrome in children, *MLR* Monocyte/lymphocyte ratio, *MPVLR* Mean platelet volume/ lymphocyte ratio, *MPVPR* Mean Platelet volume/ platelet ratio, *NLR* Neutrophil/lymphocyte ratio, *NPR* Neutrophil/ platelet ratio, *PIV* Pan-Immune-inflammation value, *PLR* Platelet/lymphocyte ratio, *SII* Systemic Immune-inflammation index, *SIRI* Systemic inflammatory response index, *WBC* White blood cell

In patients with KD, the median PLR (89.8 vs. 111.7), NLR (1.88 vs. 2.63), MPVLR (1.56 vs. 2.05), and SII (718.5 vs. 1048.8) were lower in patients with coronary artery involvement (*p* values < 0.05) (Table 3). The parameters lymphocyte, NLR, PLR, MPVLR, and SII were identified as valuable for predicting patients with KD without CALs, exhibiting high negative predictive values (85.7%, 86.1%, 87.1%, 87.5%, and 85.7%, respectively). Figure 1 and Table 2 present the ROC curves and analysis results for the most critical parameters to predict CALs. Lymphocyte count (OR: 1.56, 95% CI: 1.11–2.21) and IVIG resistance (OR: 6.26, 95% CI: 1.07–36.65) were also identified as significant predictors of CALs in the multivariate analysis (Table 4). Furthermore, there was a moderate positive correlation between lymphocyte count and duration of fever, while albumin showed a moderate negative correlation with length of stay, with statistically significant results at the 0.01 level. Coefficients for relationships are presented in Table 5.

## Discussion

The study examined how variations in the immunopathogenesis of two conditions with similar clinical manifestations are reflected in blood count parameters and their association with clinical outcomes. A notable distinction between KD and MIS-C pertains to the age at which the affected children are diagnosed. KD affects younger children, while MIS-C occurs across a wider age range, indicating different pathophysiological mechanisms. In our cohort, the NLR, MPVLR, MPVPR, and NPR indices demonstrated high sensitivity and specificity in distinguishing MIS-C from KD and predicting the need for intensive care unit admissions in MIS-C patients. Additionally, the NLR, PLR, MPVLR, and SII indices were identified as valuable markers for predicting that no coronary artery lesions might develop in KD, showing high negative predictive values. Markers such as NLR, PLR, MPVLR, MPVPR, NPR, and SIRI showed moderate correlations with the length of hospital and/or the duration of fever. From the hemogram data, SIRI and MPVLR were independent predictors of ICU admission in MIS-C, and lymphocyte count was an independent predictor of CALs in KD. The results of our study contribute to the field of clinical decision-making by elucidating the diagnostic and prognostic value of various inflammatory markers in patients diagnosed with both KD and MIS-C.

In the first year of the pandemic, an increase in diseases resembling Kawasaki Disease was noted across various countries, highlighting similarities and differences [1, 27]. MIS-C patients typically have lower lymphocyte, platelet, and albumin levels but higher CRP, ESR, ferritin, D-dimer, and cardiac markers. Furthermore, clinical phenotypes such as gastrointestinal, cardiac, neurological, and respiratory manifestations were more prevalent in MIS-C patients [2–4, 28–30]. Carter et al. [31]. found that monocyte counts in MIS-C cohorts were lower than in KD. Our study's findings were generally consistent with existing literature; however, we did not find a statistically significant difference in CRP levels. Additionally, macular rash and conjunctivitis rates were similar in MIS-C and KD in the current study. The study’s focus on MIS-C patients with KD-like features may have led to slight differences. It is essential to accurately distinguish between diseases with overlapping features to guide optimal treatment and prevent potential complications.

Beyond their role in hemostasis, platelets regulate immunity by interacting with immune cells and modulating vascular permeability, thus affecting inflammation [11]. Lower platelet distribution width and MPV have been shown in Kawasaki disease compared to healthy controls [32]. A study also determined that MPV was elevated in MIS-C patients compared to healthy controls and febrile patients hospitalized with respiratory system diseases, establishing it as a predictive biomarker for cardiac involvement [33]. Our findings are by the literature, reinforcing and corroborating existing research in this field. Platelet counts, MPV values, and MPVLR are useful for distinguishing MIS-C from KD and assessing severity. Using simple, affordable, and widely available blood tests for prognostic evaluation has the potential to save time and enhance patient care, particularly in resource-limited settings.

The predictive value of NLR, PLR, PIV, and SII in disease severity has been widely studied [20–22, 33–36]. In a study evaluating the mortality of patients with COVID-19, the values of the PIV, NLR, SII, and SIRI were significantly higher in non-survivors than in survivors [10]. Another study evaluating the hemogram of 86 newborns born to SARS-CoV-2 positive mothers found that NLR and SII were statistically significantly higher than those of newborns born to SARS-CoV-2 negative mothers [33]. Yang et al. showed that NLR and PLR could distinguish severe and non-severe COVID-19 cases with cut-off values of 3.3 and 180 [36]. A study conducted in our country found that patients with severe MIS-C have significantly higher levels of NLR and MPVLR compared to those with mild or moderate cases. However, there was no significant difference in SII levels among the different disease severities [37]. Our results cannot be thoroughly compared with previous findings due to differences in study design. The current paper highlights the effectiveness of NLR, MPVLR, MPVPR, and NPR in distinguishing between KD and MISC, as well as predicting the likelihood of intensive care admission in the MISC group. Blood cell ratios may offer better insights into systemic inflammation than individual cell counts.

A study demonstrated that PLR, NLR, MPVLR, and SII were lower, whereas lymphocyte counts were higher in KD patients with CALs compared to those without CALs. The authors also demonstrated the predictive value of SII for CALs with a cut-off value of 686 and below [38]. Similar data for indices and lymphocyte levels was observed in our cohort. However, the predictive value of SII was significant for values below 858. In line with the present study, Zheng et al. [32]. identified that platelet counts (396 vs. 324) were higher, while MPV values (9.28 vs. 9.34) were similar in the febrile period in patients with CALs. Liu et al. [39]. found that the MPV was lower in patients with KD compared to controls and that platelet counts were higher in patients with CALs than those without CALs. These data are in line with the current study. In another study, the PLR was lower in patients with CALs, which is consistent with the data from our cohort; however, unlike our findings, the NLR was higher [40]. The present study has demonstrated that lymphocyte count is an independent predictor of CALs in patients with KD. In addition, predictive thresholds for PLR, NLR, MPVLR, and SII have been determined. There is a notable difference for SII between our findings and a study evaluating 364 patients, 63% of whom had CALs [23]. The authors identified an increase in SII as an independent predictor of CALs, with an odds ratio of 1.003. Considering the possibility of developing CALs in the subacute period, their assessment of CALs at admission may partially explain the discrepancy. The results obtained in this study are reinforced by numerous studies in the literature, indicating that these parameters may serve as valuable markers. Differences in study design and demographics may explain the observed variations. In the future, comprehensive studies on more homogeneous patient groups may elucidate these differences and identify precious cut-off values.

It should be noted that this study's retrospective nature introduces certain limitations to the findings. The comparison did not consider the impact of age-related variability on the parameters. Due to sample size limitations, we did not analyze IVIG-resistant cases. A further limitation of this study is the difficulty in ruling out MIS-C in KD patients during the pandemic, which complicates the diagnostic process. All cases were evaluated meticulously from this perspective. Distinguishing between MIS-C and KD relies on SARS-CoV-2 test results and exposure history.

## Conclusion

Differentiating between MIS-C with KD-like features and classical Kawasaki disease could be challenging due to the considerable similarity in their clinical presentations. Markers such as NLR, MPVLR, MPVPR, and NPR are instrumental in differential diagnoses and predicting disease severity in MIS-C patients. Additionally, NLR, PLR, MPVLR, and SII are valuable indices for predicting CALs in KD patients. In evaluating complete blood count parameters and composite scores, SIRI and MPVLR may act as independent predictors of ICU admission in MIS-C patients, while lymphocyte levels may serve as independent predictors of CALs in patients with KD. As these markers could be obtained from routine blood tests, they represent a time-efficient, accessible, and cost-effective tools in clinical practice. Further larger-scale, prospective, and multicenter studies are required to substantiate these findings and provide a more comprehensive perspective for clinical applications.

## Data Availability

No datasets were generated or analysed during the current study.
